# The Enhanced Sensitivity of a Porous Silicon Microcavity Biosensor Based on an Angular Spectrum Using CdSe/ZnS Quantum Dots

**DOI:** 10.3390/s19224872

**Published:** 2019-11-08

**Authors:** Rui Zhou, Zhenhong Jia, Xiaoyi Lv, Xiaohui Huang

**Affiliations:** 1 School of Physical Science and Technology, Xinjiang University, Urumqi 830046, China; zrk1129@163.com; 2 School of Information Science and Engineering, Xinjiang University, Urumqi 830046, China; xiaoz813@163.com (X.L.); hxhdemail@sina.com (X.H.)

**Keywords:** porous silicon microcavity, quantum dot, angular spectrum, refractive index amplification

## Abstract

To improve the detection sensitivity of porous silicon microcavity biosensors, CdSe/ZnS quantum dots are used to label complementary DNA molecules for the refractive index amplification and angular spectrum method for detection. In this method, the TE mode laser is used as the detection light and the light source is changed into a parallel beam by collimating and expanding the beam, which illuminates the PSM surface and receives the reflected light from the PSM surface through the detector. The angle corresponding to the weakest reflected light intensity before and after the biological reaction between probe DNA and complementary DNA of different concentrations labeled by quantum dots was measured by the detector, and the relationship between the angle change before and after the biological reaction and the complementary DNA concentration labeled by quantum dots was obtained. The experimental results show that the angle change increases linearly with increasing complementary DNA concentration. The detection limit of the experiment, as determined by fitting, is approximately 36 pM. The detection limit of this method is approximately 1/300 of that without quantum dot labeling. Our method has a low cost because it does not require the use of a reflectance spectrometer, and it also demonstrates high sensitivity.

## 1. Introduction

Porous silicon (PSi) is a type of nanosilicon material with a large specific surface area, good biocompatibility, luminescence at room temperature, and an adjustable refractive index [1]. Porous silicon can be fabricated into various optical devices by electrochemical etching and photolithography technology, which can be used to fabricate high-sensitivity optical biosensors. At present, porous silicon optical biosensors with a single layer [2], double layer [3], Bragg reflector [4], microcavity [5,6], and surface grating [7,8] have been reported. Among these porous silicon sensors, a porous silicon microcavity (PSM) sensor is a one-dimensional photonic crystal containing defect states, and their reflection spectra have excellent optical properties such as high transmittance at defect peaks and narrow half-width [9,10], which results in high detection sensitivity. X. Lv et al. successfully prepared PSM devices and immobilized antibodies on PSM devices. A specific antigen‒antibody reaction was successfully detected, and the results show that the redshift of the PSM biosensor reflectance spectrum increases with increasing antigen concentration [11]. Zhang et al. prepared a PSM with a wavelength of 1555 nm on an SOI silicon wafer by double-cell electrochemical etching. The hybridization reactions of 19 base pairs of DNA were detected, and a detection limit of 43.9 nM was obtained [12].

When porous silicon is used as a biosensor for detection, the detection sensitivity usually depends on the sensitivity of the spectrometer. Therefore, it is important to select a highly sensitive detection method for the detection of low concentrations of biomolecules. According to the relationship between the defective wavelength of the PSM structure and the incident angle of incident light on the surface, the angular spectrum detection method proposed by P. Li et al. is highly sensitive [13]. By changing the incident angle of the incident light source, the change in the incident angle theta corresponding to the weakest reflected light intensity of the PSM device before and after the biological reaction is measured, and the change in the refractive index *n* caused by a biological reaction in the biosensor is obtained, thus accomplishing biological detection.

Quantum dots (QDs) have many advantages, such as good optical stability, a long fluorescence lifetime, and controllable surface properties. Surface-modified QDs have good biocompatibility and are commonly used markers for preparing high-sensitivity biosensors [14,15]. Dihydrolipoic acid (DHLA) can be used to modify the surface properties of QDs to make them water-soluble and biocompatible [16]. Modified QDs can be covalently linked with biological molecules to maintain their biological activity and detection ability [17]. QDs’ use as markers can be divided into two categories. The first is to use the fluorescence characteristics of QDs to achieve fluorescence enhancement. Dovzhenkoab et al. successfully embedded CdSe/ZnS QDs and poly(phenylenediamine) derivative (MDMO-PPV and BEHP-co-MEH-PPV) fluorescent molecules into a PSM to modulate fluorescence enhancement and bandwidth compression [18]. Y. Li et al. added QD-labeled biotin, phosphate buffer solution (PBS), and unlabeled biotin to a streptavidin-modified PSI, which proves the feasibility of porous silicon optical biosensors based on QD fluorescence labeling, and then detected SA with different concentrations; the detection limit was 100 pM [19]. The second category uses the highly refractive index characteristics of QDs to achieve refractive index amplification. Gaur et al. successfully labeled and detected small QD biotin molecules by using the shift of the single-layer reflectance spectra of porous silicon, which increased the sensitivity of QD labeling by 6-fold [2]. C. Lv et al. used QDs to couple complementary DNA to achieve refractive index amplification, and used reflection spectroscopy to detect the hybridization reactions between complementary DNA labeled with QDs and to probe the DNA. The results showed that the sensitivity of DNA detection could be increased by more than 5-fold by using QD-labeled complementary DNA [20].

In this paper, the refractive index of the reactant is amplified using QD-labeled complementary DNA of different concentrations, and the angle change before and after the hybridization reaction between probe DNA and QD-labeled complementary DNA of different concentrations in PSM devices is detected by angular spectrum detection. After the probe DNA is fixed on the PSM device, the incident light with the same wavelength as the PSM device is obliquely incident on the surface of the PSM device after collimating beam expansion, and the weakest reflected light intensity is obtained at a certain angle θ_1_. After the hybridization reaction between QD-labeled complementary DNA and probe DNA in the device, the position of the weakest reflected light intensity can be found again at angle θ_2_. The angle change caused by the biological reaction is Δθ = θ_2_ − θ_1_. This method has higher detection sensitivity than the angular spectrum method without QD labeling.

## 2. Detection Method

**Figure 1:**
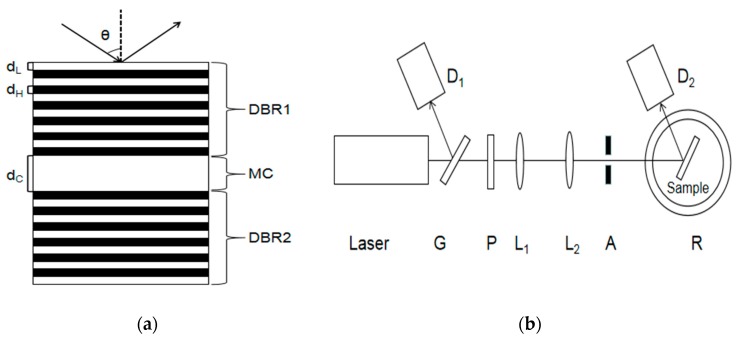
(**a**) Structural diagram of PSM device. (**b**) The schematic diagram of the experimental device.

The PSM device is composed of a Bragg mirror alternately arranged with a high and low refractive index in the upper and lower periods and a high-porosity cavity layer in the middle. Its wavelength is determined by the position of the central transmission resonance peak. The cavity layer in the middle of the PSM device is equivalent to a defect layer with high transmittance and a narrow half-width peak. If there are 25 layers of PSM devices, the central wavelength of the microcavity is 633 nm. Figure 1a shows the structure of the PSM device.

The high refractive index of the Bragg reflector is *n_H_* = 1.52, the low refractive index is *n_L_* = 1.21, and the refractive index of the intermediate cavity is *n_C_* = 1.21. The optical thickness *d_H_*, *d_L_*, and *d_C_* of the high and low refractive index and middle microcavity layer satisfy the following equations:

(1) $$n_{H}d_{H}{=n}_{L}d_{L}=\lambda_{C}/4$$

(2) $$n_{C}d_{C}=\lambda_{C}/2$$

where *n_H_*, *n_L_*, and *n_C_* represent the refractive indices of the high refractive index layer, low refractive index layer, and intermediate defect layer, respectively, and *d_H_*, *d_L_*, and *d_C_* represent the thicknesses of the high refractive index layer, low refractive index layer, and intermediate defect layer, respectively.

If the effective refractive index n of the PSM device changes, then the position of the central wavelength *λ* will also change. However, when the incident angle of the incident light source is changed to obliquely incident, the reflection spectrum of the PSM device will shift accordingly. Based on the above principle, an angular spectrum detection method is proposed. When a laser with the same wavelength as the central wavelength of the PSM is incident perpendicularly on the surface of the PSM, the reflected light intensity is the weakest. After a series of functionalization steps, the position of the central wavelength of the PSM is changed, and the weakest reflected light intensity can be obtained by deflecting the incident light at a certain angle. At this time, the angle is recorded as θ_1_. When the organism reacts in the porous silicon hole, the effective refractive index of the porous silicon device will change, deflect a certain angle θ_2_, and obtain the weakest light intensity. According to the angle change (Δθ = θ_2_ − θ_1_), the refractive index change *n* caused by a biological reaction in PSM devices can be obtained, thus realizing biological detection. Figure 1b shows the experimental device diagram.

As shown in Figure 1b, a part of the reflected light and a part of the transmitted light are obtained after the light is emitted by a semiconductor laser through a transparent glass sheet G. Detector D_1_ is used to detect the reflected light intensity and correct the influence caused by factors such as the power instability of the output light. The light passing through the glass plate is collimated and expanded by polarizer P, two thin lenses L_1_ and L_2_, and the aperture. The obtained beam is irradiated to the surface of the PSM device after passing through aperture A, and the reflected light intensity is received by detector D_2_. In this experiment, a He‒Ne laser with a divergence angle of 0.79 mrad and a wavelength of 633 nm was used as the laser source. Its wavelength is the same as the central wavelength of the PSM device.

Porous silicon can be regarded as a homogeneous mixture of silicon and air without adding any material, and its porosity is:

(3) $$\rho =\left(m_{1}-m_{2}\right)/\left(m_{1}{-m}_{3}\right)$$

where *m*_1_ is the mass before etching, *m*_2_ is the mass after etching, and *m*_3_ is the residual mass after soaking with NaOH. According to Bruggeman’s theory [21], the effective refractive index of porous silicon can be obtained via Equation (4):

(4) $$\left(1-\rho \right)\frac{n_{\mathrm{si}}{}^{2}{-n}^{2}}{n_{\mathrm{si}}{}^{2}+{2n}^{2}}+\rho \frac{1-n^{2}}{1+2n^{2}}=0$$

If something enters the hole, the equation can be transformed as follows:

(5) $$\left(1-\rho \right)\frac{n_{\mathrm{si}}{}^{2}{-n}_{0}{}^{2}}{n_{\mathrm{si}}{}^{2}+2n_{0}{}^{2}}+\left(\rho -v\right)\frac{1{-n}_{0}{}^{2}}{1+2n_{0}{}^{2}}+v\frac{n_{1}{}^{2}{-n}_{0}{}^{2}}{n_{1}{}^{2}+2n_{0}{}^{2}}=0$$

where *n*_1_ denotes the refractive index of the added material, *v* denotes the volume fraction of the material entering the hole, and *n*_0_ denotes the refractive index of the PSM device after adding the material.

**Figure 2:**
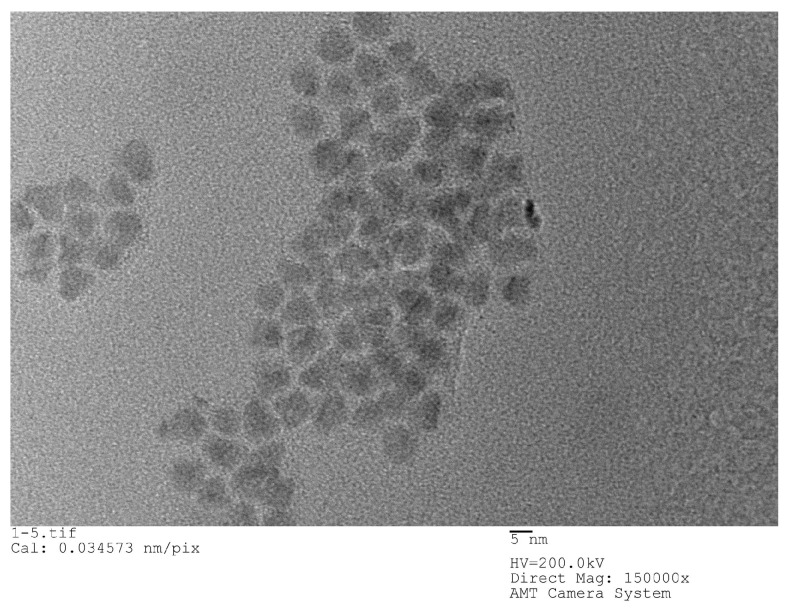
HRTEM (high-resolution transmission electron microscope) image of QDs.

When calculating the refractive index of PSM devices using QD-labeled complementary DNA and without using QD-labeled complementary DNA, the refractive index of Si is *n_si_* = 3.87, the refractive index of QD is 2.70 [22]. The QDs used in the experiments are all carboxyl water-soluble CdSe/ZnS QDs purchased from Jiayuan Quantum Dot Company (Wuhan, China). The size of QD is about 10 nm and its fluorescence peak is 625 nm, its half-width peak is within 30 nm. The structure of QDs is a CdSe core inside, which is surrounded by a ZnS shell and has a surface modified with a carboxyl group (‒COOH). Figure 2 shows an SEM image of QDs. 

**Figure 3:**
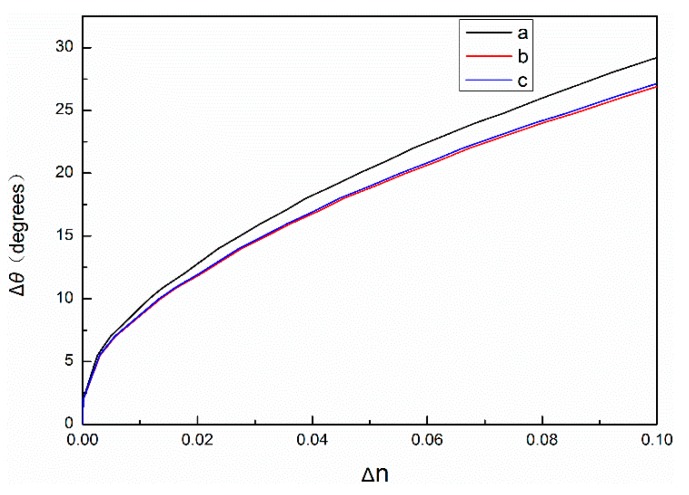
The black curve a in the figure denotes the relationship between Δθ and Δn without labeling quantum dots. The red curve b denotes the relationship between Δθ and Δn, in which the refractive index is amplified by 1.17 times. The blue curve c denotes the relationship between Δθ and Δn, in which the refractive index is amplified by 1.15 times.

The volume of a single QD is calculated as a sphere. DNA molecules are biological molecules with a refractive index of 1.33 [23,24,25]. When the probe DNA reacts with the complementary DNA, it becomes a double-helix structure with a diameter of 2 nm and a distance of 0.34 nm between adjacent base pairs. The set central wavelength of the PSM is 633 nm, and the thickness of the high refractive index layer and the low refractive index layer of the PSM device can be calculated by using Equation (1) at approximately 104 nm and 130 nm, respectively. Assuming that the first layer of the PSM device is a high refractive index layer with a porosity of 60%, each QD in the QD solution with a concentration of 15 μM can be successfully connected to a complementary DNA molecule and evenly enter the hole to connect to a probe DNA molecule. Under these conditions, the effective refractive index of PSM devices is 1.18 when the probe DNA and complementary DNA are added to PSM devices and 1.36 when the probe DNA and QD-modified complementary DNA are added; the effective refractive index of PSM devices increases by 0.18. If the layer of the PSM device is assumed to be a low refractive index layer under the same conditions and the porosity is 80%, the average number of QDs per hole is 1.2. Under these conditions, the effective refractive index of PSM devices is 1.29 when the probe DNA and complementary DNA are added to PSM devices and 1.51 when the probe DNA and QD-labeled complementary DNA are added; the refractive index of PSM devices increases by 0.22. The results show that, if the first layer is a high refractive index layer, then the refractive index of the PSM biosensor marked with QDs is 1.15 times that of the PSM biosensor without QDs. If the first layer is a low refractive index layer, the refractive index of the PSM biosensor marked with QDs is 1.17 times that of the PSM biosensor without QDs. Thus, the refractive index can be amplified by QD-labeled biomolecules participating in biological reactions. Using the above parameters, the refractive index changes are calculated by the transfer matrix method, and the calculation results are shown in Figure 3. In fact, only some of the QDs can enter porous silicon. The remaining unconnected QDs and complementary DNA will be cleaned out during subsequent processing. Therefore, due to various factors, the actual refractive index change will be smaller than the theoretical value.

As shown in Figure 3, the relationship between Δθ and Δn of PSM devices with 60% and 80% porosity and the change of angle, respectively. The refractive index of PSM devices labeled with QDs changes more when the angle changes are the same.

According to the plane wave expansion method, when the incident angle of the TE mode light wave changes, the forbidden band of PSM also changes. When the incident angle is less than 67° (θ < 67°), the incident wavelength is measurable in the forbidden band of PSM; when the incident angle continues to increase (θ > 67°), it will exceed the forbidden band [13]. Therefore, the experiment should be carried out in a small angle range.

## 3. Experiments

### 3.1. Preparation of Porous Silicon Microcavity

**Figure 4:**
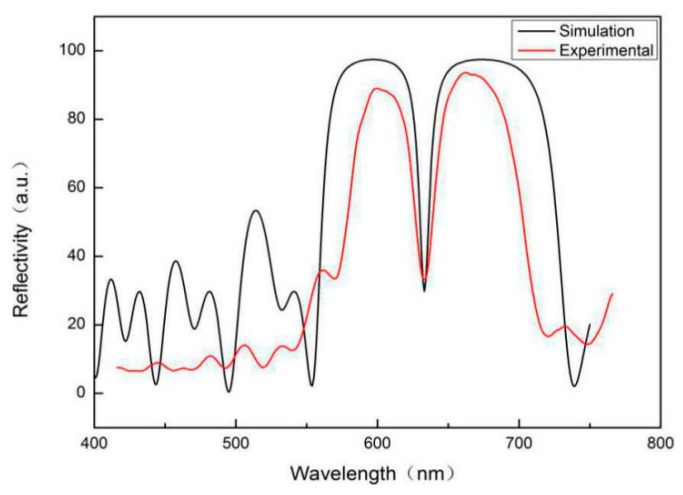
Comparison of reflection spectra between the experimental and theoretical simulation of porous silicon microcavity.

P-type boron-doped single crystal silicon (crystal orientation <100>, resistivity 0.03–0.06 Ω·cm, thickness 400 ± 10 μm) was used to fabricate PSM devices by single-channel anode electrochemical corrosion. Before the start of the experiment, the silicon wafer was cut into a square with a width of 2 cm and then cleaned with acetone, anhydrous ethanol, and deionized water in an ultrasonic cleaner for 10 min to remove impurities such as dust and grease from the surface and minimize the impact of impurities on corrosion. The cleaned silicon wafer was placed in the etching tank made of polytetrafluoroethylene. An etching solution of 40% HF solution and anhydrous ethanol (concentration ratio of 1:1) was poured into the fixed silicon wafer etching tank. According to the Labview8.0 program (National Instruments company, Austin, TX, USA), the current densities of layers with high and low refractive indices were 40 mA/cm^2^ and 90 mA/cm^2^, respectively. The etching times were 2 s and 1.7 s, respectively; the corrosion current density of the middle microcavity layer was 90 mA/cm^2^, and the control corrosion time was 3.4 s. During the corrosion process, porous silicon was formed at intervals of 3 s to ensure sufficient fluoride and corrosion uniformity. This process should be completed in a ventilated environment. The corroded porous silicon was washed with deionized water and dried in nitrogen. The reflectance spectra of the PSM devices were measured by an ultraviolet-visible spectrophotometer (Hitachi U-4100, Hitachi Ltd., Tokyo, Japan) with a resolution of 0.1 nm and compared with those calculated by the transfer matrix method, as shown in Figure 4.

In the theoretical simulation, it is generally considered that the PSM device is an ideal device without absorption and dispersion, and each dielectric interface is smooth. In the experiment, the refractive index dispersion of PSM devices will narrow the band gap of the reflection spectrum, and the absorption of light will reduce the reflectivity and increase the half-width of the defect state. In practical experiments, there will be interface fluctuations in PSM devices, rather than ideal smooth interfaces, which will reduce the reflectivity of the reflection spectrum in the forbidden band, and increase the reflectivity at the defect state and the half-width of defect state. The above factors lead to some differences between the experimental results and the theoretical simulation [26].

**Figure 5:**
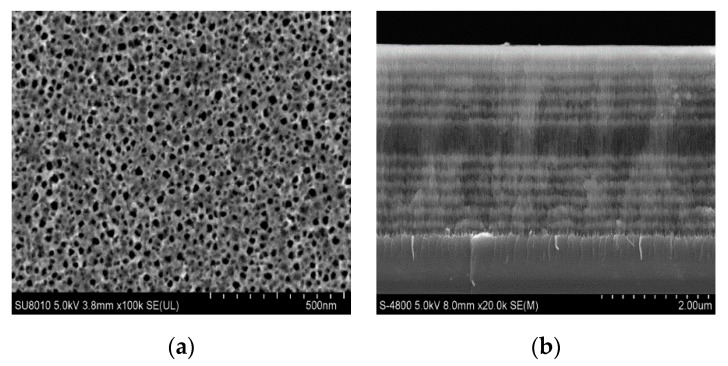
SEM of porous silicon microcavity (**a**) surface and (**b**) cross section.

Figure 5 shows a surface and cross-sectional view of a PSM device. The pore size of the prepared PSM device is approximately 30 nm, which ensures that the 16 base pairs of DNA modified by QDs (approximately 14 nm) can enter smoothly and be detected biologically. The cross section shows that the thickness of the porous silicon layer is 3.1 μm, which is consistent with the thickness of the PSM devices. The defects in each layer of the Bragg reflector and the middle layer of the PSM devices can be observed clearly.

### 3.2. Functionalization and Detection of PSM

**Figure 6:**
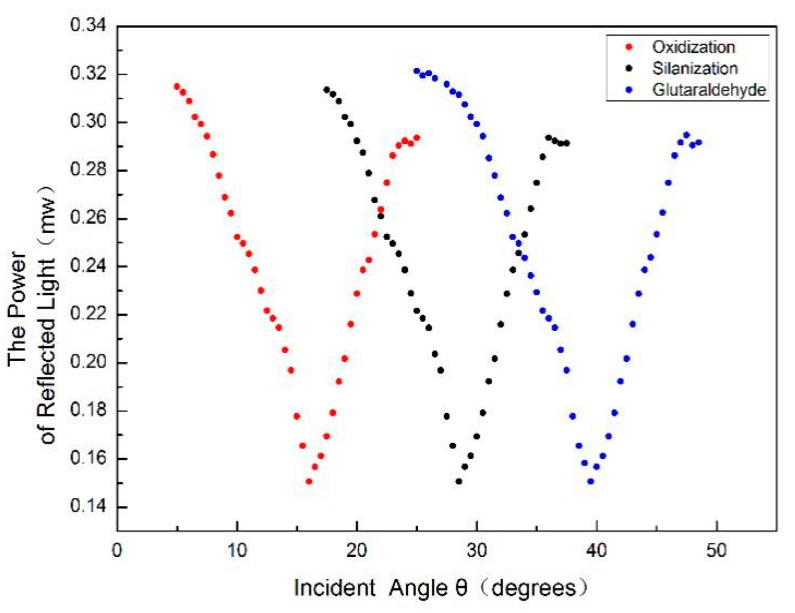
Angular spectrum contrast of the functionalization of porous silicon microcavity.

Because of the existence of Si‒H bonds on the surface of the newly prepared porous silicon, the silicon is easily oxidized in the air. Thus, it needs to be placed in 30% hydrogen peroxide solution and oxidized for 3 h at 60 °C so that a layer of SiO_2_ forms on the surface. The porous silicon was washed with deionized water and anhydrous ethanol and then cooled at room temperature. Then, the PSM device was modified with an amino group in 5% 3-aminopropyl triethoxysilane for 1 h, washed with deionized water, dried at room temperature, and then dried in a vacuum drying chamber at 100 °C for 10 min. To add the DNA molecule successfully, the silanized sample was immersed in a 2.5% glutaraldehyde solution for 1 h and then washed repeatedly with PBS and deionized water. Each step of the functionalization of PSM devices was tested by the reflection angular spectrum. Figure 6 shows a comparison of the PSM functionalization angular spectrum. The obvious angle change in the figure shows that every step of functionalization was successful.

Figure 6 clearly shows the change in angular spectrum due to the functionalization of PSM. As can be seen from the figure, the red, black and blue curves represent the PSM angle spectrum after oxidation, silanization and glutaraldehyde, respectively. The PSM angle spectrum after silanization and glutaraldehyde is red-shifted by 12.45° and 24.37°, respectively, compared with the angle spectrum after oxidation.

### 3.3. Preparation of Probe DNA and QDs-Labeled Complementary DNA

**Figure 7:**
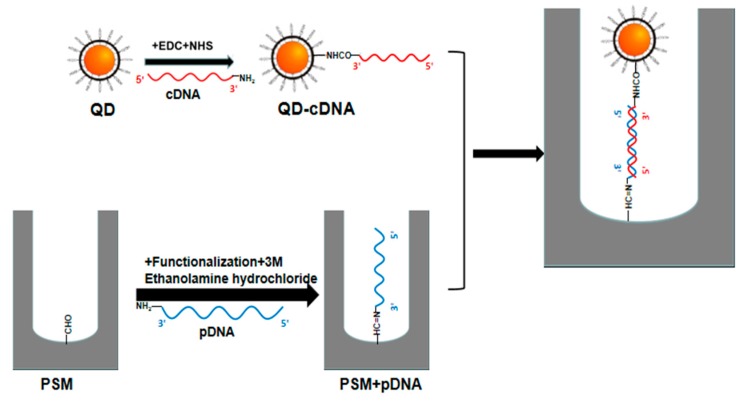
Experimental flowchart.

Probe DNA, with a volume of 30 μL and a concentration of 10 μM, was dripped onto the surface of the PSM. The probe DNA was placed in a constant-temperature box at 37 °C for 2 h. After being blown dry, the probe DNA was washed repeatedly with PBS and deionized water and then blown dry. The dried silicon wafer was immersed in 3-M ethanolamine hydrochloride and placed in a constant-temperature box at 37 °C for 1 h. After being blown dry, the silicon wafer was washed repeatedly with PBS and deionized water to ensure the full entry of probe DNA molecules into the pore. Carboxylic water-soluble CdSe/ZnS QDs with a volume of 30 μL and a concentration of 8 μM were diluted to 1 μM. Then, the carboxyl groups were activated by adding EDC and NHS at a concentration of 0.01 M and a volume of 30 μL, respectively [27]. After 30 min of the reaction at room temperature, 30 μL of complementary DNA was added at the desired concentration, light was allowed to avoid oscillation for 10 h to ensure that the QDs were fully linked with the complementary DNA, and then the sample was centrifuged for 15 min at 10,000 r/min. Complementary DNA labeled by QDs was dripped onto the PSM device with fixed probe DNA by a pipette. The device was immersed in PBS and deionized water for 2 h at 37 °C and, after extraction, it was immersed in PBS and deionized water for 15 min to remove the QDs that had not successfully connected and the complementary DNA labeled by QDs that failed to enter the hole. The preparation process of the biosensor is shown in Figure 7.

## 4. Results and Discussion

The probe DNA and complementary DNA sequences of 16 base pairs were purchased from Invitrogen Biotechnology Company (Shanghai, China); their base pair sequences are as follows:

- 5′-CAACGTTGCAGTGTAC-3′-NH2;
- 5′-GTTGCAACGTCACATG-3′-NH2.

**Figure 8:**
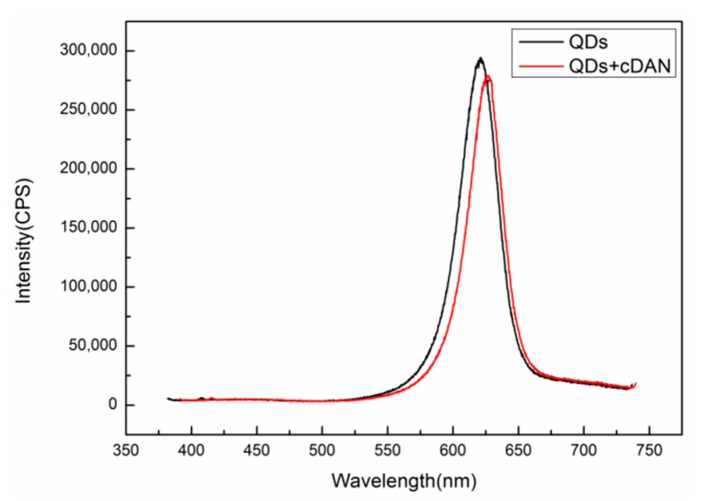
The fluorescence spectrum of QDs.

A Fluorolog-3-21-TCSPC fluorescence spectrometer (wavelength resolution 0.2 nm) was used to detect the fluorescence peaks of QDs before and after DNA coupling. The excitation wavelength is 375 nm, the excitation voltage is 700 V, and the slot width is 5 nm. The results are shown in Figure 8. The fluorescence peak of the QDs without the complementary DNA is at 623 nm, as shown in the black curve. After the QDs are coupled with complementary DNA at a concentration of 50 μM, the position of the fluorescent luminescence peak becomes 628 nm, as shown in the red curve, and the redshift is 5 nm, indicating that the QDs are successfully coupled with the complementary DNA.

**Figure 9:**
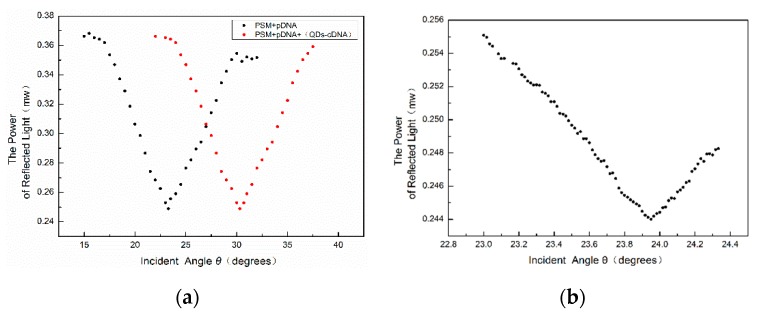
(**a**) Angular spectra of probe DNA and complementary DNA labeled by QDs before and after hybridization. The position of the weakest reflected light of the red curve in the figure is about 6.55° red-shifted compared with that of the weakest reflected light of the black curve. (**b**) Angular spectra in the range of 1.5°.

The central wavelength of PSM devices fabricated experimentally will change after the functionalization and addition of biological molecules, resulting in the inconsistency of the wavelength of the incident light. Using the oblique incidence method, the central wavelength of the PSM device can be consistent with that of the incident light source. After adding probe DNA and complementary DNA and the hybridizing reaction, the angles of the weakest reflected light intensity of PSM devices before and after the biological reaction were found by the oblique incidence method and recorded as θ_1_ and θ_2_, respectively. The angle change before and after the hybridization reaction is Δθ = θ_2_ − θ_1_. To accurately locate the position of the weakest light intensity, the intensity of each change of 1’ in the angle near the position of the weakest light intensity is recorded. The result is shown in Figure 9.

From the above figure, we can see that the position of the angular spectrum changed obviously after adding QD-labeled complementary DNA to the PSM device fixed with probe DNA. Thus, a hybridization reaction between DNA and QD-labeled complementary DNA has taken place, which enlarges the effective refractive index of the microcavity device.

**Figure 10:**
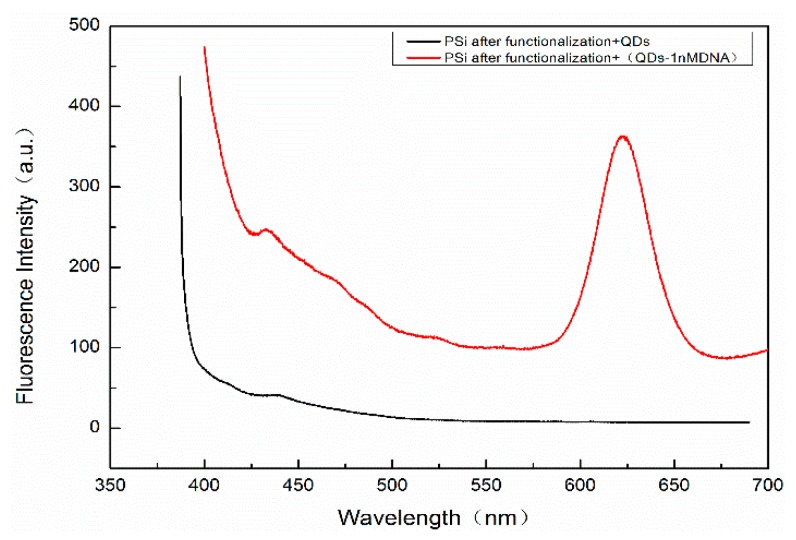
The red curve represents the fluorescence spectrum of 1 nM complementary DNA with QDs connection added to the functionalized PSM device, and the black curve represents the fluorescence spectrum with QDs added to the functionalized PSM device.

In order to detect whether there are QDs in the holes of PSM devices that have not been washed out, a Hitachi F4600 (Hitachi Ltd., Tokyo, Japan) fluorescence spectrometer (wavelength resolution is 0.1 nm) was used to detect the fluorescence peaks. The residual QDs in PSM result from two scenarios: on the one hand, QDs were not successfully coupled to the complementary DNA. On the other hand, QDs were successfully coupled with complementary DNA but failed to hybridize with the probe DNA. These QDs cannot be connected with the hole wall of porous silicon, because porous silicon is sealed by functional treatment such as oxidation, silanization, and glutaraldehyde. The result is shown in Figure 10. The excitation wavelength is 375 nm, the excitation voltage is 700 V, and the slot width is 5 nm. The fluorescence spectra of QDs dripped into fully functionalized PSM devices are shown. The absence of obvious fluorescence peaks indicates that QDs will not enter the porous silicon voids if they are not successfully linked to complementary DNA, and this will not affect the experimental results.

**Figure 11:**
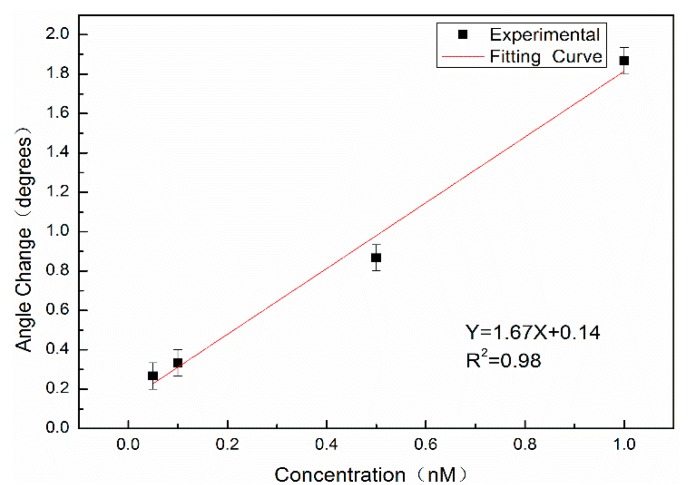
The linear relationship of DNA concentration with angle change.

Complementary DNA with concentrations of 0.05 nM, 0.10 nM, 0.50 nM, and 1.00 nM was labeled with QDs and hybridized with probe DNA; the measurable angle changes Δθ were 0.27°, 0.33°, 0.87° and 1.87°, respectively. Figure 11 shows the relationship between the angular variation and the concentration. The red line is the fitting result, where X denotes the concentration of complementary DNA labeled by QDs (nM), Y denotes the angular redshift Δθ before and after hybridization, with a slope of 1.67, and the linear correlation coefficient is *R^2^* = 0.98.

Because the surface of the PSM device fluctuates, it will cause random errors in measurement; therefore, the 3σ rule is used to calculate the experimental results. Using the same PSM blank sample, the position of the weakest reflected light intensity is measured 10 times. The standard deviation σ is calculated by Equation (6):

(6) $$\sigma =\sqrt{\frac{\sum_{i=1}^{n}{\left(Xi-\bar{X}\right)}^{2}}{\left(n-1\right)}}$$

where σ denotes the standard deviation of the position of the weakest reflected light intensity for 10 consecutive measurements, n denotes the number of measurements, and X denotes the position of the weakest reflected light intensity. The experimental results show that σ = 0.067°, and the detection limit is the concentration of complementary DNA at an angle change of 3σ, which is approximately 35.9 pM according to the linear equation.

The stability sensors and reproducibility of sensing assay are key components for monitoring of targets. Ten pieces of same functionalized porous silicon sensors have been prepared, and target DNA with concentration of 1nM has been detected for reproducibility investigation. All 10 sensors are detected by our method, and the relative standard deviation of the detection results is 4.4%. Five identical functionalized porous silicon sensors are used for sensing stability examine at five-day intervals, The DNA concentration was 1 nM and measured by proposed detection method, and the relative standard deviation of the detection results is 4.9%.

**Figure 12:**
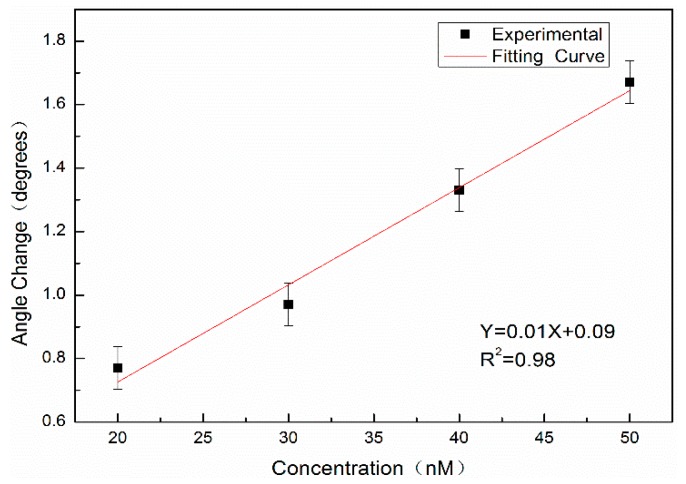
The linear fitting of the reflection angle spectrum with DNA concentration.

In addition, the complementary DNA of 20 nM, 30 nM, 40 nM, and 50 nM without QD labeling are hybridized with probe DNA at a concentration of 10 μM and detected by angular spectrum detection. The results are shown in Figure 12. The results show that the detection limit of the angular spectrum detection method for DNA molecules without QD labeling is approximately 11.1 nM.

Low detection limit can be achieved by using QDs to label biological molecules for biological detection. Y. Li et al. used QDs as markers and porous silicon biosensors to detect SA with different concentrations, and obtained a detection limit of 100 pM [19]. However, the detection limit of DNA using porous silicon biosensors without fluorescent markers is generally in the order of nM. Researchers such as Liu and Rong-xia et al. used a single-photon quantum well (pqw) structure to detect 16-base-pair DNA oligonucleotides. The experimental results showed that the detection sensitivity is 3.04 nm/muM and the detection limit is 32 nM [28]. Vilensky introduced a new type of microfluidic device that achieved a detection limit of 1 × 10^−9^ M without affecting the specificity [29].

## 5. Conclusions

Based on a p-type boron-doped PSM device, 16 base pairs of complementary DNA are successfully labeled with CdSe/ZnS water-soluble QDs to amplify the refractive index of the reactant. The hybridization reaction between probe DNA and QD-labeled complementary DNA at different concentrations is detected by angular spectrum detection using a spectrometer-free device. The results show that the angle change before and after the biological reaction is linear with the concentration of complementary DNA labeled by QDs. The detection limit of 36 pM is obtained by the angular spectrum detection method using QD labels. Compared with the angular spectrum detection method without QD labeling, the detection limit is reduced from nM to pM. The detection sensitivity of the angular spectrum detection method is improved, and the detection limit is reduced.
